# Dearth of influenza among older adults admitted with respiratory symptoms in Malaysia during the coronavirus disease 2019 pandemic in 2021

**DOI:** 10.3389/fmed.2022.977614

**Published:** 2022-10-10

**Authors:** Maw Pin Tan, Chee Loon Leong, Yong Kek Pang, Rizah Mazzuin Razali, Ahmad Izuanuddin Ismail, I-Ching Sam, Rosilawati Abdul Rani, Jennifer Chong, Mohd Arif Mohd Zim, Aisya Natasya Musa, Jia Hui Leong, Salmah Idris, Jean Khor, Adelina Cheong, Clotilde El Guerche-Séblain, Kejal Hasmukharay, Minalosani Arumugam, Khairil Erwan Khalid, Ismaliza Ismail, Wee Kooi Cheah

**Affiliations:** ^1^Department of Medicine, Faculty of Medicine, University of Malaya, Kuala Lumpur, Malaysia; ^2^Department of Medicine, Hospital Kuala Lumpur, Kuala Lumpur, Malaysia; ^3^Department of Medicine, Faculty of Medicine, Universiti Teknologi Majlis Amanah Rakyat, Sungai Buloh, Malaysia; ^4^Department of Medical Microbiology, Faculty of Medicine, University of Malaya, Kuala Lumpur, Malaysia; ^5^Clinical Research Centre, Hospital Taiping, Taiping, Malaysia; ^6^Department of Medical Microbiology, University of Malaya Medical Centre, Kuala Lumpur, Malaysia; ^7^Department of Pathology, Hospital Kuala Lumpur, Kuala Lumpur, Malaysia; ^8^Department of Medical, Sanofi Pasteur, Kuala Lumpur, Malaysia; ^9^Department of Global Vaccine Epidemiology and Modelling, Sanofi Pasteur, Singapore, Singapore; ^10^Department of Medicine, University of Malaya Medical Centre, Kuala Lumpur, Malaysia; ^11^Department of Medicine, Hospital Taiping, Perak, Malaysia

**Keywords:** influenza, COVID-19, aged, RSV, Malaysia

## Abstract

**Introduction:**

Influenza is a common respiratory virus which leads to over 400,000 annual deaths globally. Mortality from influenza is highest among those aged 75 years and over living in Africa and Southeast Asia.

**Objective:**

To determine the burden of influenza among older adults presenting to public hospitals with severe acute respiratory infection (SARI) during the coronavirus disease 2019 (COVID-19) pandemic.

**Methods:**

This multi-center, prospective, observational study recruited individuals aged 65 years and over who presented to four Malaysian hospitals with SARI from 1 January to 31 December 2021. Those with prior confirmed severe acute respiratory syndrome coronavirus-2 (SARS-CoV-2) infection were excluded. SARS-CoV-2 was detected through real-time polymerase chain reaction (PCR) with routine diagnostic kits. Influenza A, influenza B and respiratory syncytial virus (RSV) viruses were detected with Xpress Flu/RSV kits using the GeneXpert rapid real-time PCR system (Cepheid, USA).

**Results:**

Samples were obtained from 512 participants, comprising 296 (57.8%) men and 216 (42.2%) women, with a mean age (SD) of 74.0 (7.1) years. Inpatient death occurred in 48 (9.6%) individuals. Significant differences existed in age, ethnicity, and comorbidities across study sites. One (0.2%) case of influenza A, two (0.4%) cases of RSV and 63 (12.5%) cases of SARS-CoV-2 infection were detected over the 1-year period. Cases of COVID-19 mirrored national trends derived from open source data, while the dearth of influenza cases mirrored national and global Flunet figures.

**Conclusion:**

Our observational study conducted during the COVID-19 pandemic detected only one case of influenza, alongside a high SARS-CoV-2 positivity rate. The poor uptake of influenza vaccination nationally, worsened by the recent pandemic restrictions, could lead to waning immunity from the absence of seasonal exposure. Potentially deadly outbreaks may then occur when lockdown and infection control measures are eventually removed.

## Introduction

Influenza is a common respiratory infection. Prior to the coronavirus disease 2019 (COVID-19) pandemic, the median estimated number of annual influenza-associated respiratory deaths globally was 409,111 (1). The highest mortality is reported among those aged 75 years or older living in Africa and Southeast Asia (1). Up to, 67% of global influenza deaths occur in individuals aged 67 years and over (2). Clear seasonal case variations have previously been established, coinciding with the Northern and Southern Hemisphere winter seasons. Within many developed countries with temperate climates, seasonal influenza vaccines are delivered to at-risk populations and workplaces before winter (3). However, in countries with equatorial or tropical climates, the understanding of seasonality remains poor. Accurate surveillance data from these countries, which comprise mainly lower-to-middle income nations, are currently limited (4).

The severe adult respiratory syndrome coronavirus-2 (SARS-CoV-2) was first discovered in Wuhan, China, in December 2019 (5). It was initially called the novel coronavirus as it was not previously known to man. This meant humans had no natural immunity against this virus. Infection by the SARS-CoV-2 virus, therefore, led to severe pneumonia with a high mortality rate. Despite the imposition of strict lockdown measures, the virus continued to spread across all continents. The World Health Organization eventually declared the COVID-19 pandemic (6). For the subsequent 2 years, various countries imposed varying degrees of lockdown in response to spikes in cases. Governments also mandated universal masking in public places. Infection control measures, including hand hygiene, cough etiquette, and physical distancing were also enforced (7, 8). The influenza viruses, like SARS-CoV-2, are transmitted through respiratory droplets. Movement restriction, masking, and infection control measures would have, therefore, also prevented the spread of the influenza viruses as well as other respiratory viruses (9).

Global surveillance data have suggested an absence of influenza seasons in both the Northern and Southern Hemispheres over the past 2 years (10). Mandatory reporting and surveillance data for influenza are, however, limited in lower-to-middle income countries and countries with tropical climates (11). We therefore conducted a 1-year multi-center surveillance study in Malaysia, a middle-income country with a tropical climate situated in Southeast Asia, to determine the burden of influenza among older adults presenting to public hospitals with severe acute respiratory infection (SARI) during the COVID-19 pandemic.

## Materials and methods

### Study design and location

**FIGURE 1:**
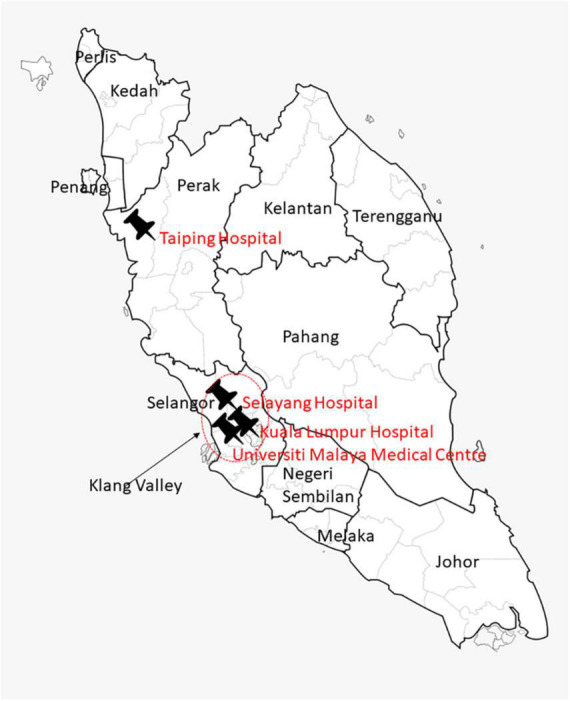
Map of Malaysia displaying the geographical locations for the four study sites. The red circle identifies the Klang Valley, the location of three of the four study sites: Universiti Malaya Medical Centre, Kuala Lumpur Hospital, and Selayang Hospital. Taiping Hospital is located in the northern state of Perak.

This was a multi-center, prospective, observational survey involving individuals aged 65 years and over. Participants were recruited from four hospitals in the Peninsular Malaysia (Figure 1). The Kuala Lumpur General Hospital (KLGH) is the largest hospital in Malaysia, with 1,855 beds. It provides secondary care to a local catchment population within the city of Kuala Lumpur. Taiping Hospital (HT) is located within the state of Perak, in northern Malaysia. The population of Taiping has a higher proportion of older persons than other states due to outward migration of younger adults to urban areas. Selayang Hospital (SH) is located in the state of Selangor and serves as a general hospital for the population of the town of Gombak and its suburban surroundings. KLGH, HT and SH are taxpayer-funded Ministry of Health hospitals, providing free medical care to those aged 60 years and over. The Universiti Malaya Medical Centre (UMMC) is a 1,500-bedded teaching hospital located between the two cities of Kuala Lumpur and Petaling Jaya. UMMC provides subsidized secondary care to local residents while also acting as a tertiary referral center.

### Study population

This study was conducted from 1 January 2021 to 31 December 2021. Individuals aged 65 years and over who met the clinical criteria of SARI, with a fever of ≥ 38°C and cough requiring hospitalization, were recruited from the participating sites through convenience sampling (12). The exclusion criteria included a positive test for SARS-CoV-2 prior to admission, a history of close contact with an individual with COVID-19, elective admissions, readmissions within 48 h of discharge from hospital, social admissions, transfers from other institutions, and inability to provide informed consent. Written informed consent was obtained prior to inclusion, and the study had obtained approval from the Malaysian Research Ethics Committee (NMRR-20-1718-55807) and the local institutional review board for UMMC (MECID-2020925-9104).

Sample size calculation was determined using an estimated expected prevalence of influenza of 3%. This was based on the low detection rate of influenza for 2020, of between 1 and 3%, due to the COVID-19 pandemic (10). A precision of 0.025 with 95% confidence was used to meet the normal distribution of data. The required sample size for this study was hence estimated as 503 after taking into consideration a 10% of screen fail (11).

### Sampling method

Nasopharyngeal and oropharyngeal swabs were obtained from participants. Samples were taken with flocked rayon swabs by trained medical personnel donning appropriate personal protective clothing. The swabs were then transported to the individual sites’ laboratories in UTM viral transport media (Copan, USA) and stored at –4°C for further analysis.

### Laboratory analysis

All swab samples underwent nucleic acid extraction and real-time polymerase chain reaction (PCR) for SARS-CoV-2 with commercial extraction and diagnostic kits routinely used in each site. All kits have received approval from the Medical Device Authority, Malaysia. For extraction, KLGH, HT and SH used RNA Isolation Kit with the KingFisher Duo Prime Purification System (Thermo Fisher Scientific, USA), Viral RNA Isolation Kit with the Liferiver EX3600 (Liferiver Biotech, China), QIAsymphony DSP Virus/Pathogen Mini Kit (Qiagen, Germany), and/or the Magcore Plus II Viral Nucleic Acid Extraction Kit (RBS Bioscience, Taiwan); PCR was performed with Real-Q 2019-nCoV Detection Kit (BioSewoom, South Korea), LyteStar 2019-nCoV RT-PCR Kit (ADT Biotech, Malaysia) and Allplex SARS-CoV-2 Assay (Seegene, South Korea). UMMC used either the GENTi Advanced DNA/RNA Extraction Kit (GeneAll, Korea) and Allplex SARS-CoV-2 Assay or the fully automated cobas SARS-CoV-2 Test on the cobas 6,800 system (Roche, USA). In addition, samples were tested for influenza A and B viruses and respiratory syncytial virus (RSV) using the Xpert Xpress Flu/RSV kits and GeneXpert rapid real-time PCR system (Cepheid, USA). Assays were performed and results were analyzed following manufacturers’ instructions. All PCR tests were performed within 48 h of sample collection.

### Data collection

Sociodemographic and clinical data were obtained from recruited participants through hospital records whenever possible or through telephone interviews. Information on age, gender, educational level, ethnicity, past medical history, and medications were recorded using a standardized data collection document. Symptoms including cough, shortness of breath, runny nose, chest pain, fever, and other complications were also recorded.

### Data analysis

Baseline sociodemographic and relevant clinical data were summarized as mean with standard deviation or frequencies and percentages for continuous variables, such as age, and for categorical variables, such as sex and medical history, respectively. Run charts were devised for aggregated monthly cases, positivity rates by age groups and total cases for all sites. Comparisons were made with national COVID-19 case detection rates, and national and international influenza surveillance data. Data on national COVID-19 case detection rates deposited by the Ministry of Health, Malaysia were obtained through an open source website^1^ (13). Age-specific population figures were obtained from the Department of Statistics of Malaysia (14). Data on national and global influenza cases were obtained from Flunet (15).

## Results

### Basic characteristics

A total of 512 nasopharyngeal and oropharyngeal samples were obtained across the four sites. Table 1 summarizes the basic characteristics of all participants recruited to this study. Significant differences in age, ethnicity, and medical history of hypertension, cardiovascular disease, and respiratory disease were observed across sites. Of the 512 recruited participants, 48 (9.6%) died during their hospital stay, 11 (2.2%) were transferred to another hospital for continuation of care, and 443 (88.2%) were discharged home.

**TABLE 1:** Basic characteristics of participants.

Participants	All sites	Individual sites				
	Total (*N* = 512)	HKL (*n* = 359, 70.1%)	HT (*n* = 86, 16.8%)	UMMC (*n* = 57, 11.1%)	SH (*n* = 10, 2.0%)	*P*-value
Age (yrs), mean (SD)	74.0 (7.1)	73.2 (6.6)	75.1 (7.4)	76.3 (8.7)	75.9 (5.5)	0.001*
**Age group (yrs), *n* (%)**						
65–74	311 (60.7)	236 (65.7)	42 (48.8)	28 (49.1)	5 (50.0)	0.007*
75–84	154 (30.1)	98 (27.3)	34 (39.5)	19 (33.3)	3 (30.0)	
85 and over	47 (9.2)	25 (7.0)	10 (11.6)	10 (17.5)	2 (20.0)	
**Gender, *n* (%)**						
Male	296 (57.8)	206 (57.4)	50 (58.1)	34 (59.6)	6 (60.0)	0.992
Female	216 (42.2)	153 (42.6)	36 (41.9)	23 (40.4)	4 (40.0)	
**Ethnicity, *n* (%)**						
Chinese	158 (30.9)	116 (32.3)	17 (19.8)	22 (30.9)	3 (30.0)	0.020*
Indian and others	100 (19.5)	68 (18.9)	14 (16.3)	16 (28.1)	2 (20.0)	
Malay	254 (49.6)	175 (48.7)	55 (64.0)	19 (33.3)	5 (50.0)	
**Underlying comorbidities, *n* (%)**						
Diabetes	262 (51.2)	186 (51.8)	46 (53.5)	26 (45.6)	4 (40.0)	0.709
Hypertension	253 (49.4)	220 (61.3)	17 (19.8)	16 (28.1)	0	<0.001*
Cardiovascular disease	203 (39.6)	127 (35.4)	44 (51.2)	27 (47.4)	5 (50.0)	0.022*
Respiratory disease	117 (22.9)	89 (24.8)	12 (14.0)	11 (19.3)	5 (50.0)	0.025*

*Significant at *p* < 0.05. HKL, Hospital Kuala Lumpur; HT, Hospital Taiping; UMMC, University of Malaya Medical Centre; SH, Selayang Hospital; SD, standard deviation.

### Influenza virus detection

Table 2 provides the total numbers and percentages of nasopharyngeal and oropharyngeal samples which tested positive for influenza, RSV and SARS-CoV-2 by each quarter of 2021. Overall, only one (0.2%) case of influenza A and two (0.4%) cases of RSV were detected out of the total 512 samples. However, 63 (12.5%) samples tested positive for SARS-CoV-2. SARS-CoV-2 positive cases occurred throughout the year, with a spike in the third quarter of July to September 2021. It was not possible to determine trends for influenza and RSV cases due to the low number of cases identified (Table 2).

**TABLE 2:** Number of cases and positivity rates across sites.

Months, 2021	*N*	No. of cases (%)		
		Influenza A	RSV	SARS-CoV-2
January–March	151	0 (0.0)	1 (0.7)	6 (4.1)
April–June	118	0 (0.0)	0 (0.0)	11 (9.8)
July–September	150	0 (0.0)	1 (0.7)	38 (25.3)
October–December	93	1 (1.1)	0 (0.0)	8 (8.6)
Total	512	1 (0.2)	2 (0.4)	63 (12.3)

RSV, respiratory syncytial virus; SARS-CoV-2, severe acute respiratory syndrome coronavirus-2.

### Comparisons with coronavirus disease 2019 cases and FluNet

**FIGURE 2:**
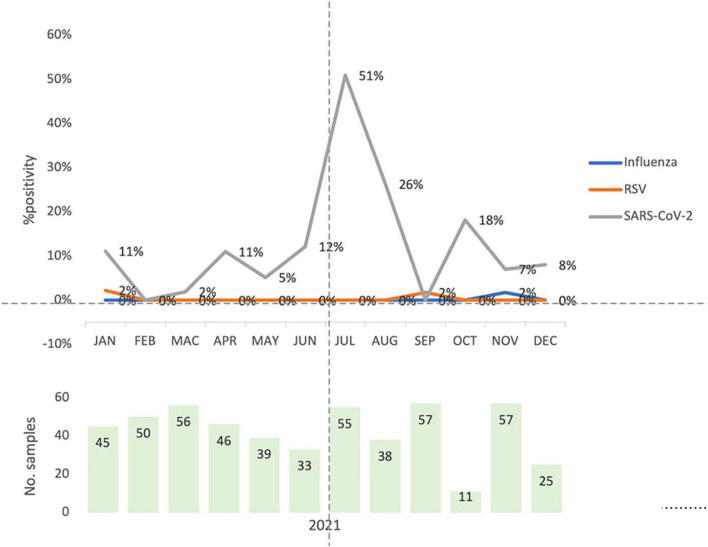
Total monthly virus detection rates for influenza, respiratory syncytial virus and severe acute respiratory syndrome coronavirus-2 for all four study sites. The overall monthly percentage positivity rates for influenza virus (influenza, blue), respiratory syncytial virus (RSV, orange) and the severe acute respiratory syndrome coronavirus-2 (SARS-CoV-2, gray) are shown cumulatively for all four study sites for the surveillance study. The light green bars represent the total number of samples obtained for each month.

Figure 2 shows the positivity rates for influenza, RSV and SARS-CoV-2 during each month of recruitment. Eleven to 57 samples were obtained each month, with positivity rates of 0% in February and September and 51% in July for SARS-CoV-2. One case of influenza was detected in November 2021, while two cases of RSV were detected overall, one in January 2021 and one in September 2021.

**FIGURE 3:**
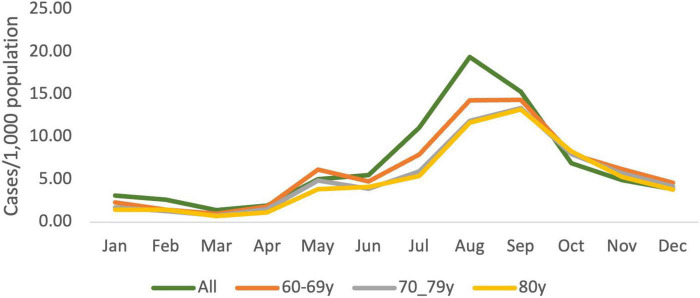
Monthly newly detected cases of COVID-19 in Malaysia for the total population and by age groups for 2021. Total numbers of new cases and the number of new cases for all ages (green) and individuals within the age groups of 60–69 (orange), 70-79 (gray) and 80 (yellow) years reported in Malaysia per 1,000 population each month in 2021. Data obtained from https://github.com/MoH-Malaysia/covid19-public (13).

In comparison, the total number of new cases of COVID-19 per 1,000 population reported nationally peaked in August 2021 (Figure 3). Positivity rates for SARS-CoV-2 detection were also freely available for the age groups of 60–69, 70–79, and 80 years and above. The number of new cases per 1,000 population was similar for all three age groups and reflected the trend for the overall population.

**FIGURE 4:**
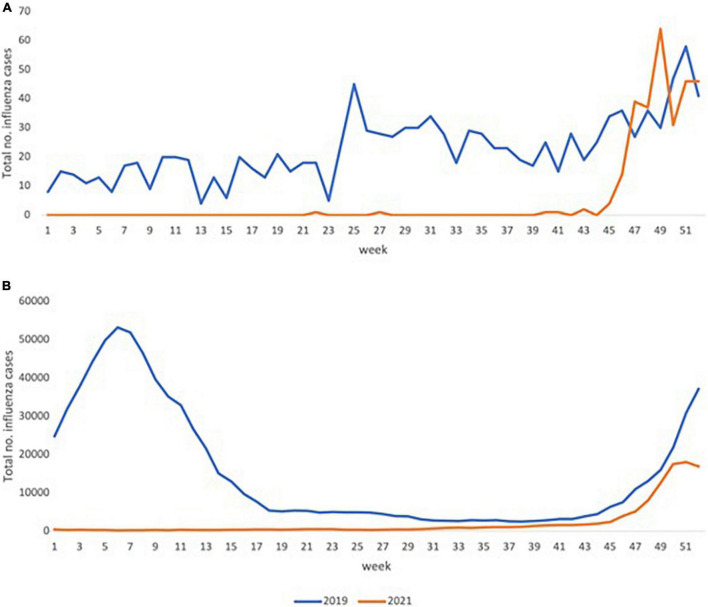
Total number of cases of influenza reported for Malaysia and globally according to Flunet surveillance for 2019 and 2021. The total number of influenza cases reported to the World Health Organization web-based tool for influenza virological surveillance (FluNet). The above figures depict the total number of weekly reported cases for Malaysia **(A)** and globally **(B)** for the years 2019 (blue) and 2021 (orange). *Data obtained from FluNet* (15).

Figure 4 shows the FluNet surveillance data for the total number of influenza cases reported from Malaysia and globally. The total cases of influenza reported in Malaysia was zero for all but two of the weeks for the first 39 weeks of the year 2021. The number of reported cases started increasing at the 45th week. Global trends were nearly identical to Malaysian trends, with virtually no cases reported up to week 31, followed by a gradual rise between weeks 31 and 43 and a sharp rise thereafter.

## Discussion

This influenza surveillance study was conducted in three hospitals within the Klang Valley and one hospital north-west Malaysia. A near-absence of influenza and RSV infections in 2021 was apparent throughout the 12-month study period. The mean SARS-CoV-2 positivity rate was high at 12.3% during the same period. This finding was aligned with the timing of decreased influenza circulation in Malaysia reported through FluNet, which is a web-based influenza surveillance system hosted by the World Health Organization (16). Molecular diagnostic tests for influenza are rarely performed on adults with respiratory symptoms and influenza is not considered a notifiable disease, leading to a scarcity of reliable data on influenza burden in developing countries in Southeast Asia (17).

As reported in other studies, SARS-CoV-2 appears to have become the dominant respiratory viral illness requiring hospital admissions in adults in many countries. Winter spikes in COVID-19 cases are now apparent in the northern hemisphere for 2 years in a row. This suggests that COVID-19 has become endemic, and may take on seasonality (10). Lockdown, physical distancing and infection control measures intended to curb the spread of SARS-CoV-2 have also effectively wiped out influenza virus infections in many countries in 2020 and 2021 (18–20). However, it is unlikely that travel restrictions, universal masking and physical distancing will continue indefinitely. Furthermore, the spikes observed in the last 10 weeks of 2021 do suggest that the influenza virus is still present (14, 15). For RSV, which mainly affects children, historically low rates were reported in the winter seasons of both Australia (southern hemisphere) and the USA (northern hemisphere) between October 2019 and September 2021, with unusual summer spikes (21). This disruption of typical RSV circulation has also been attributed to COVID-19 control measures.

The influenza virus has posed a continual threat to human hosts though its ability to survive in populations with considerable prior exposure. This is achieved through antigenic drift with resultant immune escape. Annual vaccinations are, therefore, necessary to address the constantly changing vaccine targets. The development of annual vaccines are informed by a global influenza surveillance system (22). The sporadic case detection of influenza during the COVID-19 pandemic may potentially challenge influenza vaccine strain selection. It has also been suggested that the drastic reduction in cases of seasonal influenza infection globally may lead to a reduction in population immunity (23). A rebound in the number of influenza cases may occur with the lifting of travel restrictions and masking recommendations. The dip in immunity may then lead to increased disease severity. The need for annual vaccination programs for influenza, particularly in high-risk populations such as older adults and healthcare workers should, therefore, continue to be advocated to minimize the impact of loss of immunity to the influenza virus (24).

Prior to the pandemic, the burden of respiratory viruses such as influenza and RSV among adults was under-recognized in many developing countries (25). The gold standard diagnosis test for respiratory viruses is PCR, which is expensive and not widely available. Those presenting to secondary care with viral pneumonia are, therefore, often treated empirically for bacterial infections with the assumption that virus diseases are self-limiting (26). The COVID-19 pandemic has led to major investments in diagnostic laboratory capacity for PCR testing. In addition, affordable household and point-of-care testing technology has become widely accepted. The increased viral testing capacity could potentially be used to enhance the diagnostic capacity for influenza viruses and other respiratory viruses in the future (27–29). Influenza illness may not necessarily be self-limiting, but instead has been recognized as potentially life-threatening, particularly in vulnerable groups such as older adults. Antiviral use in influenza infection can reduce illness duration, severity and complication rates. Treatment effectiveness, however, is only observed with early initiation of antivirals. Early diagnostic approaches are, therefore, required to facilitate early antiviral treatment (30–32). Positive identification of the disease will inform the initiation of infection control measures to control spread. In addition, viral identification will also help ensure the delivery of adequate supportive treatment and clinical observation to reduce the risk of complications (33). This will also facilitate timely initiation of treatment for secondary infection, thrombotic events and other established complications of influenza infection (34).

The single case of influenza detected in November 2021 does reflect global trends. Globally, positivity rates based on the number of samples processed were below 1% until epidemiological week 41, peaking at 6% in weeks 50 and 51. The total number of samples obtained or processed was not available for Malaysia. The pattern of respiratory viruses presenting as SARI in Malaysia, therefore, mirrors world-wide patterns. This suggests that even with the absence of widespread travel, Malaysia continues to exhibit a similar trend in influenza infections compared to temperate climates (35). This should also add to emerging evidence that influenza infections are not a problem exclusive to temperate climates (36).

Our study utilized convenience sampling and involved three sites located in the Greater Klang Valley and one site in the northern region. The findings of this study may, therefore, not be representative of the national picture. Indeed, national trends of COVID-19 infection suggest that spikes do tend to emerge from the Greater Klang Valley prior to spikes in other states. Nevertheless, the absence of influenza infections during the pandemic does follow international patterns and will provide important data with which to inform national vaccination policies. Alongside the issues with COVID-19 vaccine inequality, influenza vaccination programs for vulnerable groups such as older persons do not currently exist in many countries in Africa and Southeast Asia, where influenza deaths have been greatest prior to the COVID-19 pandemic (37). This should be taken into consideration in global strategies to address the impending spike in influenza infections that now threatens recovery once travel restrictions and infection control measures for COVID-19 are no longer in place.

## Conclusion

A 1-year surveillance study was conducted in four public hospitals among individuals aged 65 years and over presenting with SARI in a middle-income developing country during the COVID-19 pandemic. A near absence of influenza cases was observed during 2021. While SARS-CoV-2 now appears to be the dominant transmissible respiratory virus leading to hospital admissions, infection control measures and travel restrictions are also effective in preventing transmission of the influenza virus. Concerns now exist for a reduction in natural immunity to the influenza virus with the potential threat of a rebound in influenza cases once pandemic restrictions are no longer in place. With the absence of influenza vaccination programs in developing nations, this now presents a greater threat to the recovery of developing nations from the COVID-19 pandemic. Emphasis should also be placed on an accurate surveillance strategy, which may include mandatory reporting as well as public availability of data.

## Data availability statement

The datasets presented in this article are not readily available because aggregated data is available on request from WKC, CRC Taiping. Requests to access the datasets should be directed to WKC.

## Ethics statement

The studies involving human participants were reviewed and approved by the Malaysian Research Ethics Committee (NMRR-20-1718-55807) and University of Malaya Medical Centre (MECID-2020925-9104). The patients/participants provided their written informed consent to participate in this study.

## Author contributions

MPT, CLL, ICS, YKP, RMR, AII, JK, AC, CEG-S, and WKC conceived, designed, and obtained funding for the study. JHL, II, SI, RAR, MAMZ, ANM, KH, and MA were involved with participant recruitment, data collection, sample collection, and data analysis. JC was involved with coordination of sample collection and sample analysis. MPT, ICS, RAR, KH, and WKC were involved with data analysis. MPT, CLL, ICS, YKP, RMR, AII, JHL, and WKC drafted the manuscript. All authors were involved with editing of the manuscript and reviewed and approved the final manuscript.

## Footnotes

1
https://github.com/MoH-Malaysia/covid19-public
